# Supplementary material for: Gold Glyconanoparticles Combined with 91–99 Peptide of the Bacterial Toxin, Listeriolysin O, Are Efficient Immunotherapies in Experimental Bladder Tumors
Source: Cancers (Basel). 2022 May 13;14(10):2413. doi: 10.3390/cancers14102413 (PMC9140107; doi:10.3390/cancers14102413)
Supplement: Supplementary file 1 [file cancers-14-02413-s001.zip › cancers-1653970-supplementary.pdf]

Gold glyconanoparticles combined to 91-99 peptide of the bacterial toxin, listeriolysin O, are efficient immunotherapies in experimental bladder tumor

Table S1. Cytokine pattern in sera of patients with different solid tumors.

Patients code[a]	Clinical clasification	Treatments	IL-17A[b,*]	IFN-	IL-2	IL-6	IL-10	TNF-
MEL-1	Metastatic melanoma (IV)	NT	84.7 ± 0.1	3.1 ± 0.1	15.58 ± 0.4	33.4 ± 0.9	23.6 ± 0.9	45.25 ± 0.1
MEL-2	Nodular melanoma (III B)	Tumor surgery	105 ± 0.5	2.5± 0.1	11.47 ± 0.3	19.1 ±0.1	19.6± 0.1	13.78± 0.1
MEL-3	Metastatic melanoma (IV)	NT	89.5 ± 0.5	2.3 ± 0.1	19.65 ± 0.2	19.3 ± 0.2	13.3 ± 9.2	13.25 ± 0.1
BC-1	Lung and bladder carcinoma	Cisplastin-ectoposide	16.32± 0.1	1.4± 0.1	2.22 ± 0.2	6.0± 0.1	4.6± 0.1	7.8 ± 0.1
BC-2	Urothelial bladder carcinoma	NT	2.21 ± 1.2	0.9 ± 0.1	0.8 ± 0.1	12.1 ± 0.4	2.35 ± 0.3	17.61± 0.2
BC-3	Urothelial bladder carcinoma	NT	6.4 ± 0.2	1.9 ± 0.1	1.29± 0.1	6.07 ± 0.2	8.14 ± 0.2	18.3 ± 0.1
HEP-1	Hepatocellular carcinoma	Ablation by microwaves	18.5± 0.1	2.1± 0.1	3.36 ± 0-3	19± 0.9	6.4± 0.1	3.84± 0.1
PROST-1	Prostate adenocarcinoma	Taxocel	11.5 ± 0.1	4.4± 0.2	4.14 ± 0.3	6.6± 0.2	4.1± 0.2	6.8 ± 0.1
GLIO-1	Multiform glioblastoma	Temozolamide-radiotherapy	12.2 ± 0.3	1.4 ± 0.2	4.93 ± 0.2	8.8±0.8	3.88 ± 0.2	12.2 ± 0.2
CONT-1	NONE	NT	2.8 ± 0.1	2.4± 0.1	3.0 ± 0.2	3.1± 0.1	2.4± 0.1	2.0± 0.1
CONT-2	NONE	NT	3.5 ± 0.1	2.3± 0.1	3.15 ± 0.2	3± 0.1	2.3± 0.1	2.1± 0.1

[a] Clinical manifestations and treatments of patients with informed consent selected for the study. CONT, healthy donors. [b] Cytokines are measured in sera of patients (Luminex kits, EMD Millipore Corporation, Billerica MA). Results are the mean of cytokine concentrations (pg/mL) ± SD. (* $p < 0.05$).

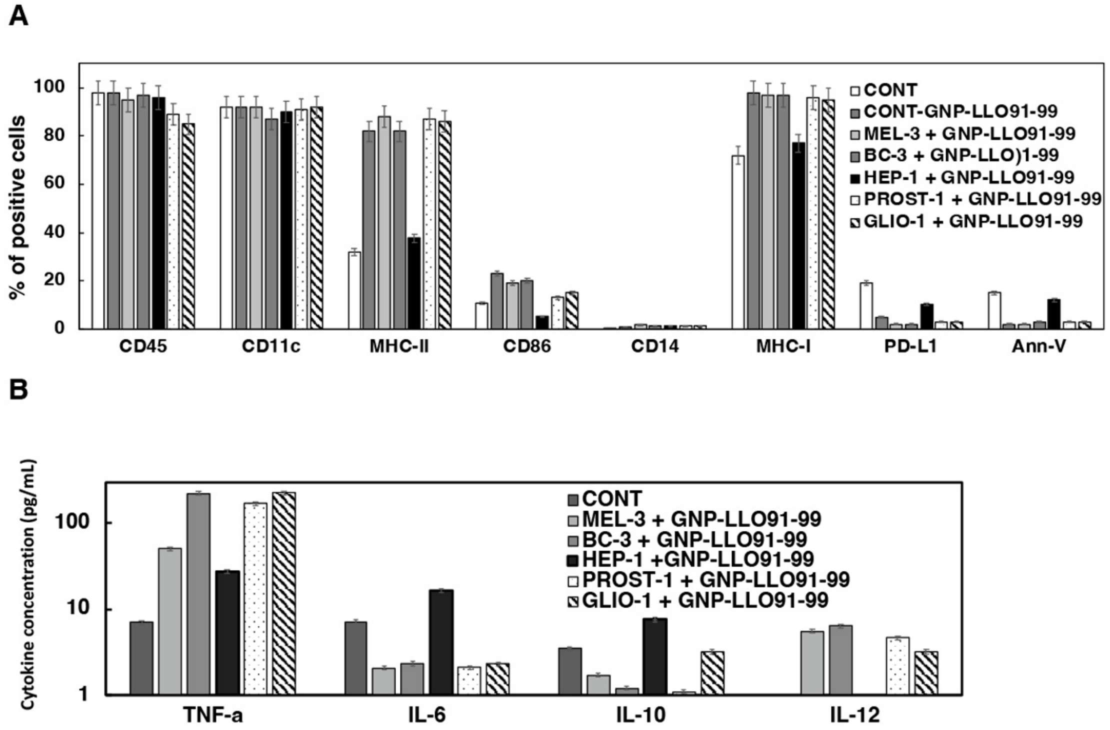

Figure S1. GNP-LLO$_{91-99}$ nanovaccines effect as adjuvants in MoDC from human donors. (**a**) MoDC from healthy donors (CONT), melanoma (MEL-3), urothelial bladder cancer (BC-3), hepatocellular carcinoma (HEP-1), prostate adenocarcinoma (PROST-1) and multiforme glioblastoma (GLIO-1) patients were incubated with GNP-LLO$_{91-99}$ (50 µg/mL) for 16 hours. MoDC were analysed for DC (CD45, CD11c), monocyte (CD14), antigen-presentation (MHC-I, MHC-II, CD86) and cell-death (PD-L1 or Annexin-V) surface markers by FACS using specific monoclonal antibodies. Results are expressed as the mean percentages of positive cells ± SD ($P \leq 0.5$). (**b**) Supernatants of same MoDC as in (**a**) after GNP-LLO$_{91-99}$ treatment were collected and cytokine concentration analysed (Luminex kits, EMD Millipore Corporation, Billerica MA). Results are the mean of cytokine concentrations (pg/mL) ± SD. (*$P < 0.5$).

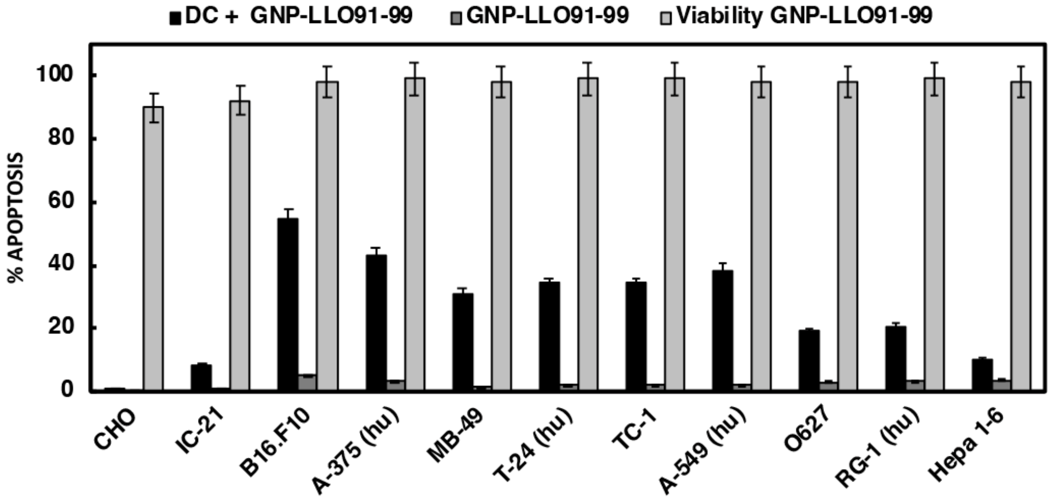

Figure S2. GNP-LLO$_{91-99}$ nanovaccines effect onto cell toxicity, direct apoptosis, and immunogenic apoptosis of solid tumors. Hamster CHO ovary tumor, murine IC-21 macrophage-like tumor cells, murine B16.F10 and human A-375 melanoma, murine MB-49 and human T-24 bladder tumors, murine TC-1, and human A-549 NSCLC lung tumors, murine O627 and human RG-1 glioblastoma and murine Hepa 1-6 hepatocellular carcinoma were incubated 16 hours at 37ºC with GNP-LLO$_{91-99}$ nanovaccines (50 μg/mL) and examined for viability tests using Trypan blue staining (light grey bars) or for direct apoptosis using flow cytometry (black bars). For immunogenic apoptosis, tumors are incubated with ½ supernatants of DC/MoDC pre-treated with GNP-LLO$_{91-99}$ nanovaccines (50 μg/mL for 16 hours at 37ºC) (dark grey bars). Apoptosis was examined by FACS using the DNA marker, 7-AAD (7-AAD-PE) and the apoptotic marker, annexin V (annexin V-APC). Results for cell viability are expressed as the mean of unstained cells ± SD ($P < 0.5$). Results for apoptosis are expressed as percentages of apoptotic cells ± SD ($P \leq 0.5$). GNP-LLO$_{91-99}$ nanovaccines caused no cell toxicity or direct apoptosis into tumor cells, while they were able to induce immunogenic apoptosis of hot tumors as melanoma (B16.F10, A-375), lung NSCLC tumors (TC-1, A-549), bladder tumors (MB-49) or cold tumors as glioblastoma (O627, RG-1) but not in other cold tumors as ovary tumors (CHO), SV-40 induced macrophage-like tumor cells (IC-21) or hepatocellular carcinoma (Hepa 1-6).
